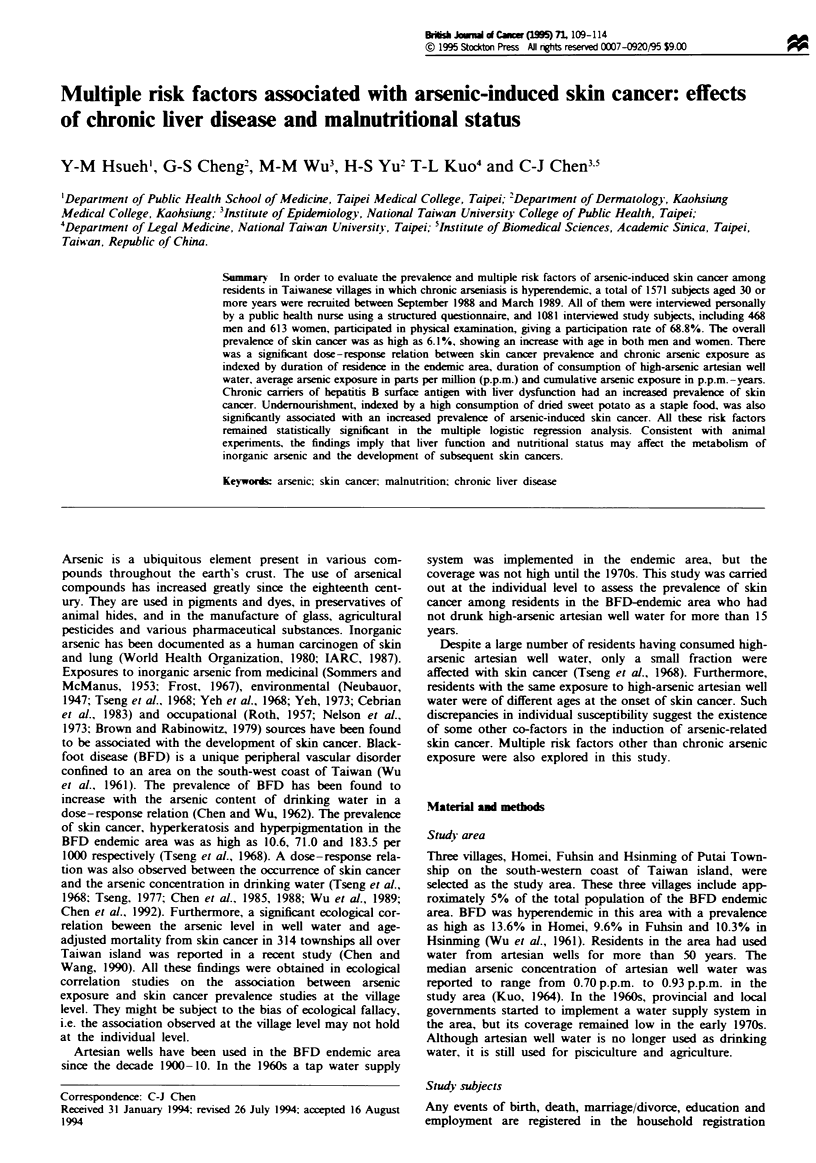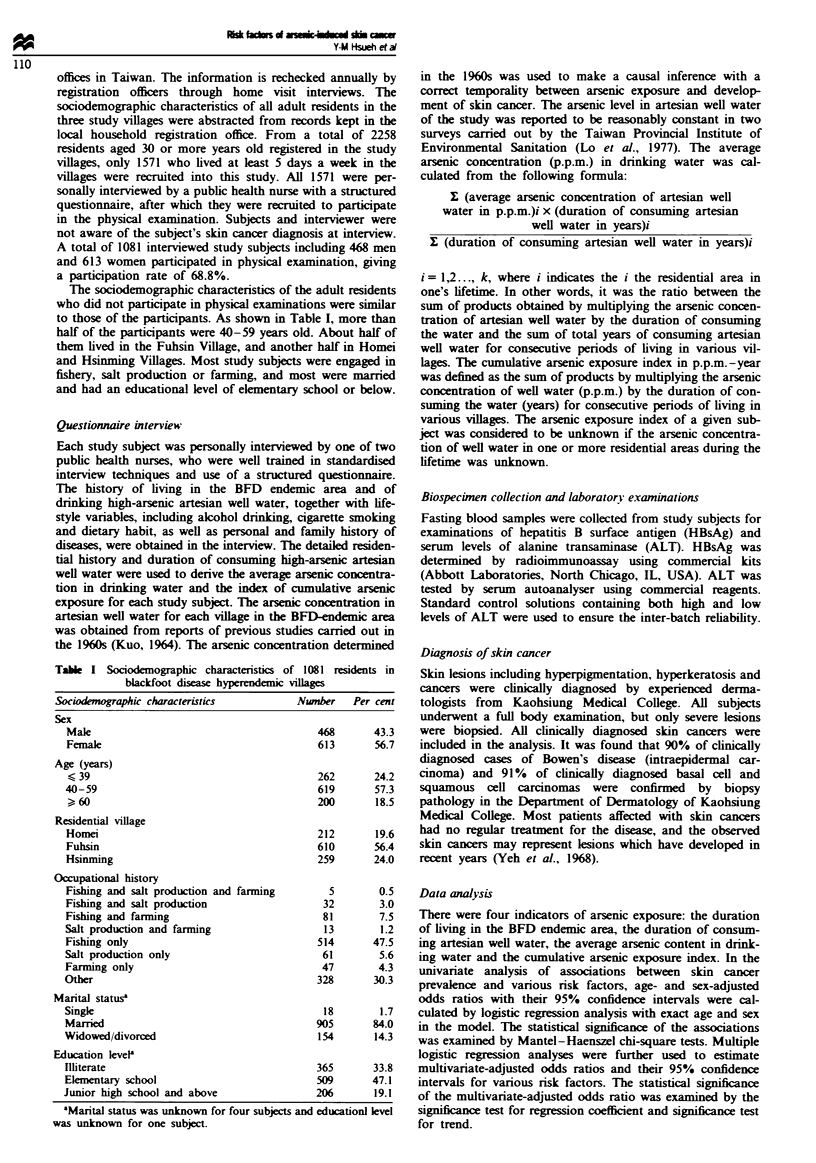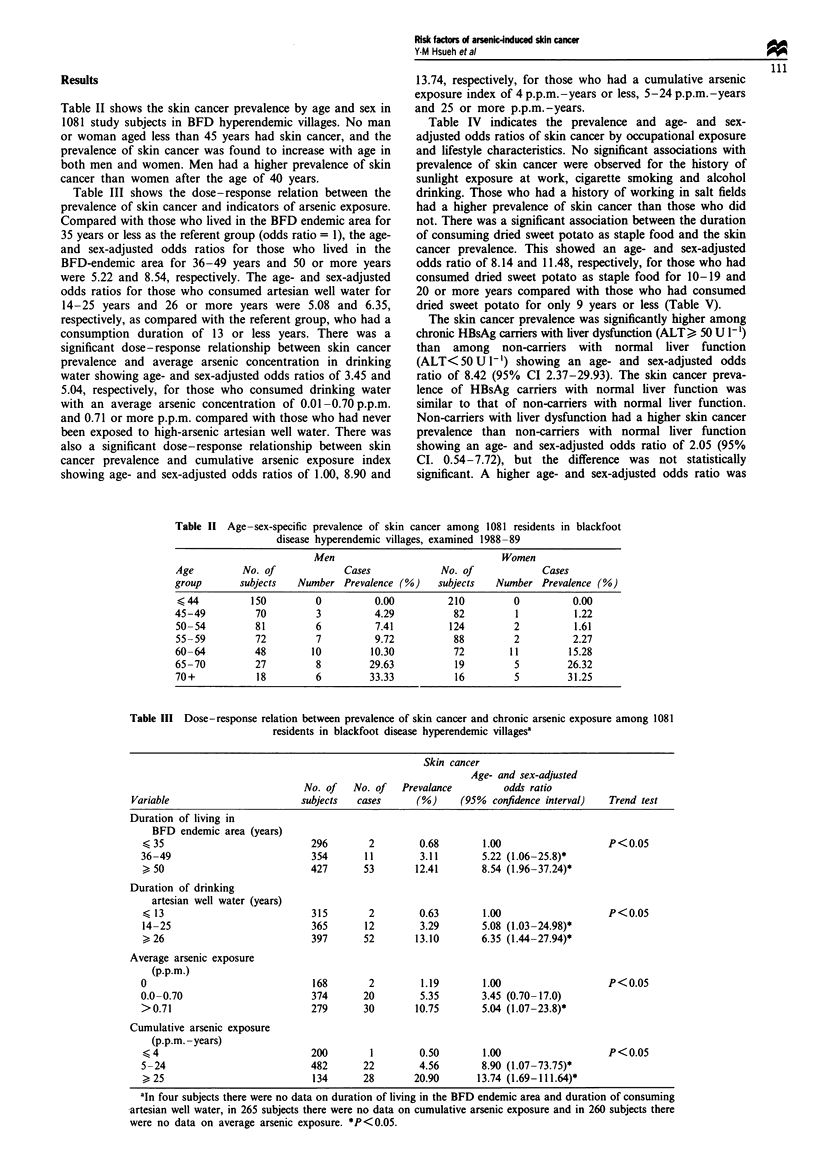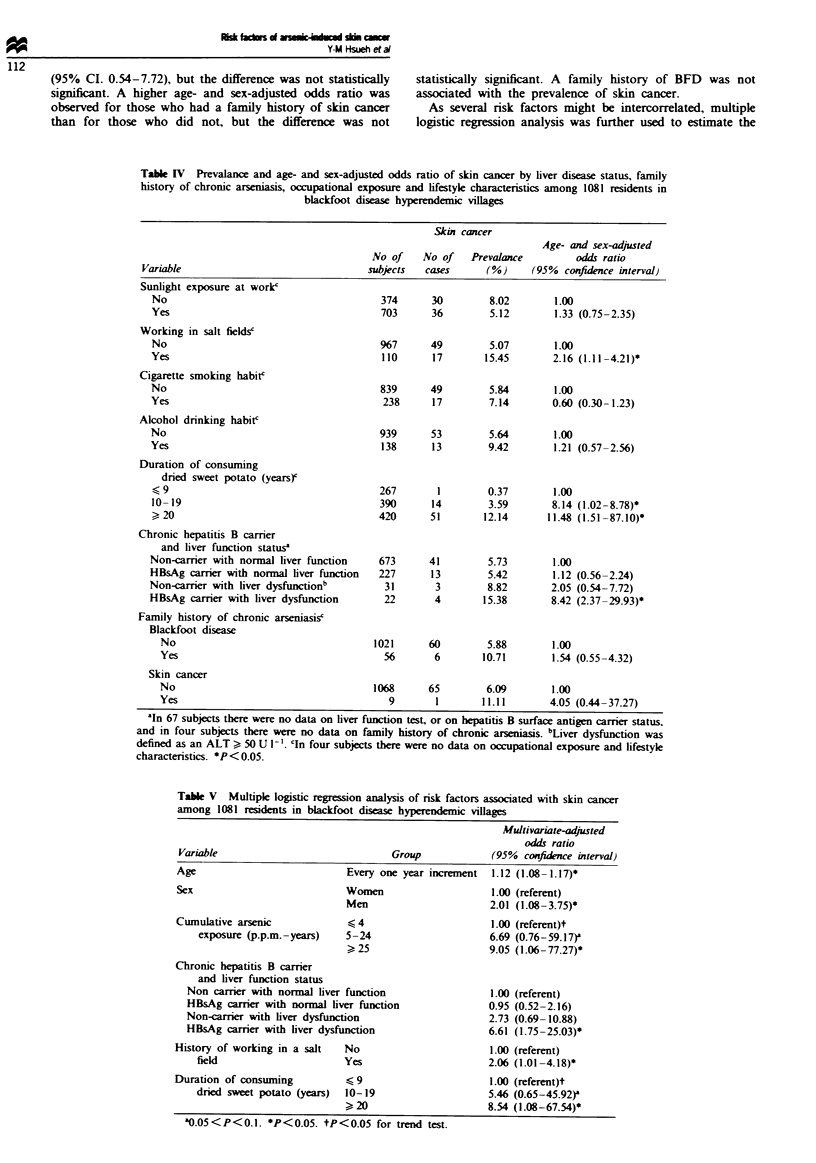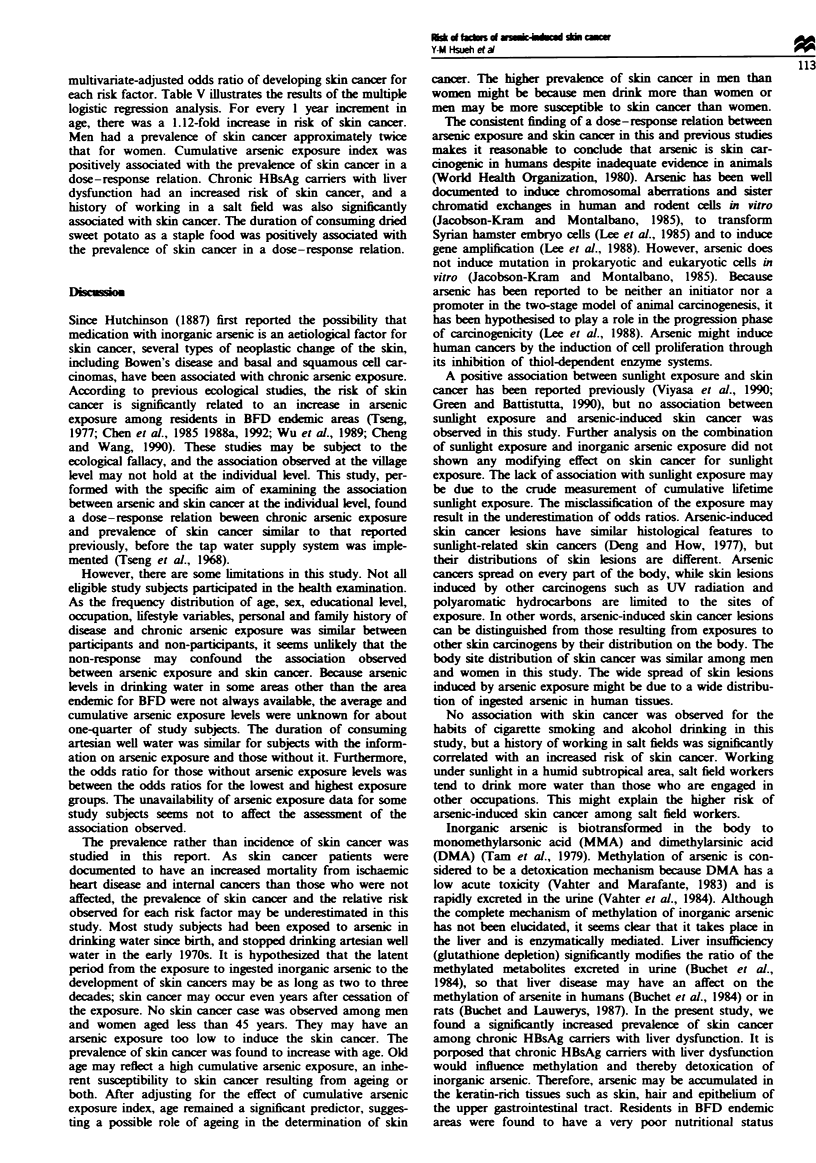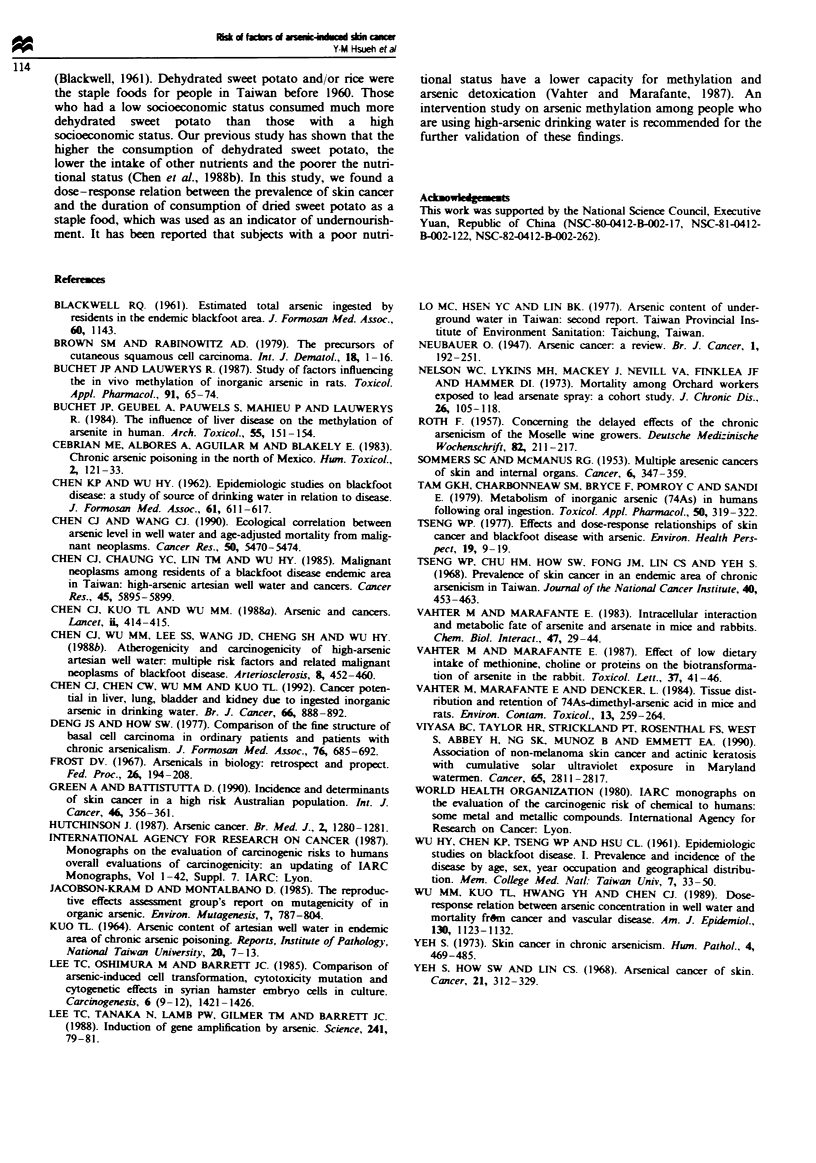# Multiple risk factors associated with arsenic-induced skin cancer: effects of chronic liver disease and malnutritional status.

**DOI:** 10.1038/bjc.1995.22

**Published:** 1995-01

**Authors:** Y. M. Hsueh, G. S. Cheng, M. M. Wu, H. S. Yu, T. L. Kuo, C. J. Chen

**Affiliations:** Department of Public Health School of Medicine, Taipei Medical College, Taiwan, Republic of China.

## Abstract

In order to evaluate the prevalence and multiple risk factors of arsenic-induced skin cancer among residents in Taiwanese villages in which chronic arseniasis is hyperendemic, a total of 1571 subjects aged 30 or more years were recruited between September 1988 and March 1989. All of them were interviewed personally by a public health nurse using a structured questionnaire, and 1081 interviewed study subjects, including 468 men and 613 women, participated in physical examination, giving a participation rate of 68.8%. The overall prevalence of skin cancer was as high as 6.1%, showing an increase with age in both men and women. There was a significant dose-response relation between skin cancer prevalence and chronic arsenic exposure as indexed by duration of residence in the endemic area, duration of consumption of high-arsenic artesian well water, average arsenic exposure in parts per million (p.p.m.) and cumulative arsenic exposure in p.p.m.-years. Chronic carriers of hepatitis B surface antigen with liver dysfunction had an increased prevalence of skin cancer. Undernourishment, indexed by a high consumption of dried sweet potato as a staple food, was also significantly associated with an increased prevalence of arsenic-induced skin cancer. All these risk factors remained statistically significant in the multiple logistic regression analysis. Consistent with animal experiments, the findings imply that liver function and nutritional status may affect the metabolism of inorganic arsenic and the development of subsequent skin cancers.


© 1995 Stockton Press All rights reserved 0007-0920/95 $9.00

### Multiple risk factors associated with arsenic-induced skin cancer: effects of chronic liver disease and malnutritional status

Y-M Hsueh[1], G-S Cheng[2], M-M Wu[3], H-S Yu[2] T-L Kuo[4] and C-J Chen[3,5]

[1]*Department of Public Health School of Medicine, Taipei Medical College, Taipei;* [2]*Department of Dermatology, Kaohsiung Medical College, Kaohsiung;* [3]*Institute of Epidemiology, National Taiwan University College of Public Health, Taipei;* [4]*Department of Legal Medicine, National Taiwan University, Taipei;* [5]*Institute of Biomedical Sciences, Academic Sinica, Taipei, Taiwan, Republic of China.*

**Summary** In order to evaluate the prevalence and multiple risk factors of arsenic-induced skin cancer among residents in Taiwanese villages in which chronic arseniasis is hyperendemic, a total of 1571 subjects aged 30 or more years were recruited between September 1988 and March 1989. All of them were interviewed personally by a public health nurse using a structured questionnaire, and 1081 interviewed study subjects, including 468 men and 613 women, participated in physical examination, giving a participation rate of 68.8%. The overall prevalence of skin cancer was as high as 6.1%, showing an increase with age in both men and women. There was a significant dose-response relation between skin cancer prevalence and chronic arsenic exposure as indexed by duration of residence in the endemic area, duration of consumption of high-arsenic artesian well water, average arsenic exposure in parts per million (p.p.m.) and cumulative arsenic exposure in p.p.m.-years. Chronic carriers of hepatitis B surface antigen with liver dysfunction had an increased prevalence of skin cancer. Undernourishment, indexed by a high consumption of dried sweet potato as a staple food, was also significantly associated with an increased prevalence of arsenic-induced skin cancer. All these risk factors remained statistically significant in the multiple logistic regression analysis. Consistent with animal experiments, the findings imply that liver function and nutritional status may affect the metabolism of inorganic arsenic and the development of subsequent skin cancers.

**Keywords:** arsenic; skin cancer; malnutrition; chronic liver disease

Arsenic is a ubiquitous element present in various compounds throughout the earth's crust. The use of arsenical compounds has increased greatly since the eighteenth century. They are used in pigments and dyes, in preservatives of animal hides, and in the manufacture of glass, agricultural pesticides and various pharmaceutical substances. Inorganic arsenic has been documented as a human carcinogen of skin and lung (World Health Organization, 1980; IARC, 1987). Exposures to inorganic arsenic from medicinal (Sommers and McManus, 1953; Frost, 1967), environmental (Neubauor, 1947; Tseng *et al.*, 1968; Yeh *et al.*, 1968; Yeh, 1973; Cebrian *et al.*, 1983) and occupational (Roth, 1957; Nelson *et al.*, 1973; Brown and Rabinowitz, 1979) sources have been found to be associated with the development of skin cancer. Blackfoot disease (BFD) is a unique peripheral vascular disorder confined to an area on the south-west coast of Taiwan (Wu *et al.*, 1961). The prevalence of BFD has been found to increase with the arsenic content of drinking water in a dose-response relation (Chen and Wu, 1962). The prevalence of skin cancer, hyperkeratosis and hyperpigmentation in the BFD endemic area was as high as 10.6, 71.0 and 183.5 per 1000 respectively (Tseng *et al.*, 1968). A dose-response relation was also observed between the occurrence of skin cancer and the arsenic concentration in drinking water (Tseng *et al.*, 1968; Tseng, 1977; Chen *et al.*, 1985, 1988; Wu *et al.*, 1989; Chen *et al.*, 1992). Furthermore, a significant ecological correlation beween the arsenic level in well water and age-adjusted mortality from skin cancer in 314 townships all over Taiwan island was reported in a recent study (Chen and Wang, 1990). All these findings were obtained in ecological correlation studies on the association between arsenic exposure and skin cancer prevalence studies at the village level. They might be subject to the bias of ecological fallacy, i.e. the association observed at the village level may not hold at the individual level.

Artesian wells have been used in the BFD endemic area since the decade 1900-10. In the 1960s a tap water supply system was implemented in the endemic area, but the coverage was not high until the 1970s. This study was carried out at the individual level to assess the prevalence of skin cancer among residents in the BFD-endemic area who had not drunk high-arsenic artesian well water for more than 15 years.

Despite a large number of residents having consumed high-arsenic artesian well water, only a small fraction were affected with skin cancer (Tseng *et al.*, 1968). Furthermore, residents with the same exposure to high-arsenic artesian well water were of different ages at the onset of skin cancer. Such discrepancies in individual susceptibility suggest the existence of some other co-factors in the induction of arsenic-related skin cancer. Multiple risk factors other than chronic arsenic exposure were also explored in this study.

### Material and methods

### *Study area*

Three villages, Homei, Fuhsin and Hsinming of Putai Township on the south-western coast of Taiwan island, were selected as the study area. These three villages include approximately 5% of the total population of the BFD endemic area. BFD was hyperendemic in this area with a prevalence as high as 13.6% in Homei, 9.6% in Fuhsin and 10.3% in Hsinming (Wu *et al.*, 1961). Residents in the area had used water from artesian wells for more than 50 years. The median arsenic concentration of artesian well water was reported to range from 0.70 p.p.m. to 0.93 p.p.m. in the study area (Kuo, 1964). In the 1960s, provincial and local governments started to implement a water supply system in the area, but its coverage remained low in the early 1970s. Although artesian well water is no longer used as drinking water, it is still used for pisciculture and agriculture.

### *Study subjects*

Any events of birth, death, marriage/divorce, education and employment are registered in the household registration

Correspondence: C-J Chen
Received 31 January 1994; revised 26 July 1994; accepted 16 August 1994

offices in Taiwan. The information is rechecked annually by registration officers through home visit interviews. The sociodemographic characteristics of all adult residents in the three study villages were abstracted from records kept in the local household registration office. From a total of 2258 residents aged 30 or more years old registered in the study villages, only 1571 who lived at least 5 days a week in the villages were recruited into this study. All 1571 were personally interviewed by a public health nurse with a structured questionnaire, after which they were recruited to participate in the physical examination. Subjects and interviewer were not aware of the subject's skin cancer diagnosis at interview. A total of 1081 interviewed study subjects including 468 men and 613 women participated in physical examination, giving a participation rate of 68.8%.

The sociodemographic characteristics of the adult residents who did not participate in physical examinations were similar to those of the participants. As shown in Table I, more than half of the participants were 40–59 years old. About half of them lived in the Fuhsin Village, and another half in Homei and Hsinming Villages. Most study subjects were engaged in fishery, salt production or farming, and most were married and had an educational level of elementary school or below.

*Questionnaire interview*

Each study subject was personally interviewed by one of two public health nurses, who were well trained in standardised interview techniques and use of a structured questionnaire. The history of living in the BFD endemic area and of drinking high-arsenic artesian well water, together with lifestyle variables, including alcohol drinking, cigarette smoking and dietary habit, as well as personal and family history of diseases, were obtained in the interview. The detailed residential history and duration of consuming high-arsenic artesian well water were used to derive the average arsenic concentration in drinking water and the index of cumulative arsenic exposure for each study subject. The arsenic concentration in artesian well water for each village in the BFD-endemic area was obtained from reports of previous studies carried out in the 1960s (Kuo, 1964). The arsenic concentration determined in the 1960s was used to make a causal inference with a correct temporality between arsenic exposure and development of skin cancer. The arsenic level in artesian well water of the study was reported to be reasonably constant in two surveys carried out by the Taiwan Provincial Institute of Environmental Sanitation (Lo *et al.*, 1977). The average arsenic concentration (p.p.m.) in drinking water was calculated from the following formula:

$$\frac{\Sigma\ (\text{average arsenic concentration of artesian well water in p.p.m.})i \times (\text{duration of consuming artesian well water in years})i}{\Sigma\ (\text{duration of consuming artesian well water in years})i}$$

$i = 1,2\ldots,\ k$, where $i$ indicates the $i$ the residential area in one's lifetime. In other words, it was the ratio between the sum of products obtained by multiplying the arsenic concentration of artesian well water by the duration of consuming the water and the sum of total years of consuming artesian well water for consecutive periods of living in various villages. The cumulative arsenic exposure index in p.p.m.-year was defined as the sum of products by multiplying the arsenic concentration of well water (p.p.m.) by the duration of consuming the water (years) for consecutive periods of living in various villages. The arsenic exposure index of a given subject was considered to be unknown if the arsenic concentration of well water in one or more residential areas during the lifetime was unknown.

**Table I** Sociodemographic characteristics of 1081 residents in blackfoot disease hyperendemic villages

| *Sociodemographic characteristics* | *Number* | *Per cent* |
|---|---|---|
| Sex | | |
| Male | 468 | 43.3 |
| Female | 613 | 56.7 |
| Age (years) | | |
| ⩽ 39 | 262 | 24.2 |
| 40–59 | 619 | 57.3 |
| ⩾ 60 | 200 | 18.5 |
| Residential village | | |
| Homei | 212 | 19.6 |
| Fuhsin | 610 | 56.4 |
| Hsinming | 259 | 24.0 |
| Occupational history | | |
| Fishing and salt production and farming | 5 | 0.5 |
| Fishing and salt production | 32 | 3.0 |
| Fishing and farming | 81 | 7.5 |
| Salt production and farming | 13 | 1.2 |
| Fishing only | 514 | 47.5 |
| Salt production only | 61 | 5.6 |
| Farming only | 47 | 4.3 |
| Other | 328 | 30.3 |
| Marital status[a] | | |
| Single | 18 | 1.7 |
| Married | 905 | 84.0 |
| Widowed/divorced | 154 | 14.3 |
| Education level[a] | | |
| Illiterate | 365 | 33.8 |
| Elementary school | 509 | 47.1 |
| Junior high school and above | 206 | 19.1 |

[a]Marital status was unknown for four subjects and educationl level was unknown for one subject.

*Biospecimen collection and laboratory examinations*

Fasting blood samples were collected from study subjects for examinations of hepatitis B surface antigen (HBsAg) and serum levels of alanine transaminase (ALT). HBsAg was determined by radioimmunoassay using commercial kits (Abbott Laboratories, North Chicago, IL, USA). ALT was tested by serum autoanalyser using commercial reagents. Standard control solutions containing both high and low levels of ALT were used to ensure the inter-batch reliability.

*Diagnosis of skin cancer*

Skin lesions including hyperpigmentation, hyperkeratosis and cancers were clinically diagnosed by experienced dermatologists from Kaohsiung Medical College. All subjects underwent a full body examination, but only severe lesions were biopsied. All clinically diagnosed skin cancers were included in the analysis. It was found that 90% of clinically diagnosed cases of Bowen's disease (intraepidermal carcinoma) and 91% of clinically diagnosed basal cell and squamous cell carcinomas were confirmed by biopsy pathology in the Department of Dermatology of Kaohsiung Medical College. Most patients affected with skin cancers had no regular treatment for the disease, and the observed skin cancers may represent lesions which have developed in recent years (Yeh *et al.*, 1968).

*Data analysis*

There were four indicators of arsenic exposure: the duration of living in the BFD endemic area, the duration of consuming artesian well water, the average arsenic content in drinking water and the cumulative arsenic exposure index. In the univariate analysis of associations between skin cancer prevalence and various risk factors, age- and sex-adjusted odds ratios with their 95% confidence intervals were calculated by logistic regression analysis with exact age and sex in the model. The statistical significance of the associations was examined by Mantel–Haenszel chi-square tests. Multiple logistic regression analyses were further used to estimate multivariate-adjusted odds ratios and their 95% confidence intervals for various risk factors. The statistical significance of the multivariate-adjusted odds ratio was examined by the significance test for regression coefficient and significance test for trend.

### Results

Table II shows the skin cancer prevalence by age and sex in 1081 study subjects in BFD hyperendemic villages. No man or woman aged less than 45 years had skin cancer, and the prevalence of skin cancer was found to increase with age in both men and women. Men had a higher prevalence of skin cancer than women after the age of 40 years.

Table III shows the dose–response relation between the prevalence of skin cancer and indicators of arsenic exposure. Compared with those who lived in the BFD endemic area for 35 years or less as the referent group (odds ratio = 1), the age- and sex-adjusted odds ratios for those who lived in the BFD-endemic area for 36–49 years and 50 or more years were 5.22 and 8.54, respectively. The age- and sex-adjusted odds ratios for those who consumed artesian well water for 14–25 years and 26 or more years were 5.08 and 6.35, respectively, as compared with the referent group, who had a consumption duration of 13 or less years. There was a significant dose–response relationship between skin cancer prevalence and average arsenic concentration in drinking water showing age- and sex-adjusted odds ratios of 3.45 and 5.04, respectively, for those who consumed drinking water with an average arsenic concentration of 0.01–0.70 p.p.m. and 0.71 or more p.p.m. compared with those who had never been exposed to high-arsenic artesian well water. There was also a significant dose–response relationship between skin cancer prevalence and cumulative arsenic exposure index showing age- and sex-adjusted odds ratios of 1.00, 8.90 and 13.74, respectively, for those who had a cumulative arsenic exposure index of 4 p.p.m.–years or less, 5–24 p.p.m.–years and 25 or more p.p.m.–years.

Table IV indicates the prevalence and age- and sex-adjusted odds ratios of skin cancer by occupational exposure and lifestyle characteristics. No significant associations with prevalence of skin cancer were observed for the history of sunlight exposure at work, cigarette smoking and alcohol drinking. Those who had a history of working in salt fields had a higher prevalence of skin cancer than those who did not. There was a significant association between the duration of consuming dried sweet potato as staple food and the skin cancer prevalence. This showed an age- and sex-adjusted odds ratio of 8.14 and 11.48, respectively, for those who had consumed dried sweet potato as staple food for 10–19 and 20 or more years compared with those who had consumed dried sweet potato for only 9 years or less (Table V).

The skin cancer prevalence was significantly higher among chronic HBsAg carriers with liver dysfunction (ALT$\geqslant$ 50 U $l^{-1}$) than among non-carriers with normal liver function (ALT$<$50 U $l^{-1}$) showing an age- and sex-adjusted odds ratio of 8.42 (95% CI 2.37–29.93). The skin cancer prevalence of HBsAg carriers with normal liver function was similar to that of non-carriers with normal liver function. Non-carriers with liver dysfunction had a higher skin cancer prevalence than non-carriers with normal liver function showing an age- and sex-adjusted odds ratio of 2.05 (95% CI. 0.54–7.72), but the difference was not statistically significant. A higher age- and sex-adjusted odds ratio was

**Table II** Age–sex-specific prevalence of skin cancer among 1081 residents in blackfoot disease hyperendemic villages, examined 1988–89

| | Men | | | Women | | |
|---|---|---|---|---|---|---|
| *Age group* | *No. of subjects* | *Cases Number* | *Cases Prevalence (%)* | *No. of subjects* | *Cases Number* | *Cases Prevalence (%)* |
| ⩽44 | 150 | 0 | 0.00 | 210 | 0 | 0.00 |
| 45–49 | 70 | 3 | 4.29 | 82 | 1 | 1.22 |
| 50–54 | 81 | 6 | 7.41 | 124 | 2 | 1.61 |
| 55–59 | 72 | 7 | 9.72 | 88 | 2 | 2.27 |
| 60–64 | 48 | 10 | 10.30 | 72 | 11 | 15.28 |
| 65–70 | 27 | 8 | 29.63 | 19 | 5 | 26.32 |
| 70+ | 18 | 6 | 33.33 | 16 | 5 | 31.25 |

**Table III** Dose–response relation between prevalence of skin cancer and chronic arsenic exposure among 1081 residents in blackfoot disease hyperendemic villages[a]

| | Skin cancer | | | | |
|---|---|---|---|---|---|
| *Variable* | *No. of subjects* | *No. of cases* | *Prevalance (%)* | *Age- and sex-adjusted odds ratio (95% confidence interval)* | *Trend test* |
| Duration of living in BFD endemic area (years) | | | | | |
| ⩽35 | 296 | 2 | 0.68 | 1.00 | $P<0.05$ |
| 36–49 | 354 | 11 | 3.11 | 5.22 (1.06–25.8)* | |
| ⩾50 | 427 | 53 | 12.41 | 8.54 (1.96–37.24)* | |
| Duration of drinking artesian well water (years) | | | | | |
| ⩽13 | 315 | 2 | 0.63 | 1.00 | $P<0.05$ |
| 14–25 | 365 | 12 | 3.29 | 5.08 (1.03–24.98)* | |
| ⩾26 | 397 | 52 | 13.10 | 6.35 (1.44–27.94)* | |
| Average arsenic exposure (p.p.m.) | | | | | |
| 0 | 168 | 2 | 1.19 | 1.00 | $P<0.05$ |
| 0.0–0.70 | 374 | 20 | 5.35 | 3.45 (0.70–17.0) | |
| >0.71 | 279 | 30 | 10.75 | 5.04 (1.07–23.8)* | |
| Cumulative arsenic exposure (p.p.m.-years) | | | | | |
| ⩽4 | 200 | 1 | 0.50 | 1.00 | $P<0.05$ |
| 5–24 | 482 | 22 | 4.56 | 8.90 (1.07–73.75)* | |
| ⩾25 | 134 | 28 | 20.90 | 13.74 (1.69–111.64)* | |

[a]In four subjects there were no data on duration of living in the BFD endemic area and duration of consuming artesian well water, in 265 subjects there were no data on cumulative arsenic exposure and in 260 subjects there were no data on average arsenic exposure. *$P<0.05$.

(95% CI. 0.54–7.72), but the difference was not statistically significant. A higher age- and sex-adjusted odds ratio was observed for those who had a family history of skin cancer than for those who did not, but the difference was not statistically significant. A family history of BFD was not associated with the prevalence of skin cancer.

As several risk factors might be intercorrelated, multiple logistic regression analysis was further used to estimate the

**Table IV** Prevalance and age- and sex-adjusted odds ratio of skin cancer by liver disease status, family history of chronic arseniasis, occupational exposure and lifestyle characteristics among 1081 residents in blackfoot disease hyperendemic villages

| Variable | No of subjects | Skin cancer: No of cases | Skin cancer: Prevalance (%) | Age- and sex-adjusted odds ratio (95% confidence interval) |
|---|---|---|---|---|
| Sunlight exposure at work[c] | | | | |
| No | 374 | 30 | 8.02 | 1.00 |
| Yes | 703 | 36 | 5.12 | 1.33 (0.75–2.35) |
| Working in salt fields[c] | | | | |
| No | 967 | 49 | 5.07 | 1.00 |
| Yes | 110 | 17 | 15.45 | 2.16 (1.11–4.21)* |
| Cigarette smoking habit[c] | | | | |
| No | 839 | 49 | 5.84 | 1.00 |
| Yes | 238 | 17 | 7.14 | 0.60 (0.30–1.23) |
| Alcohol drinking habit[c] | | | | |
| No | 939 | 53 | 5.64 | 1.00 |
| Yes | 138 | 13 | 9.42 | 1.21 (0.57–2.56) |
| Duration of consuming dried sweet potato (years)[c] | | | | |
| ⩽9 | 267 | 1 | 0.37 | 1.00 |
| 10–19 | 390 | 14 | 3.59 | 8.14 (1.02–8.78)* |
| ⩾20 | 420 | 51 | 12.14 | 11.48 (1.51–87.10)* |
| Chronic hepatitis B carrier and liver function status[a] | | | | |
| Non-carrier with normal liver function | 673 | 41 | 5.73 | 1.00 |
| HBsAg carrier with normal liver function | 227 | 13 | 5.42 | 1.12 (0.56–2.24) |
| Non-carrier with liver dysfunction[b] | 31 | 3 | 8.82 | 2.05 (0.54–7.72) |
| HBsAg carrier with liver dysfunction | 22 | 4 | 15.38 | 8.42 (2.37–29.93)* |
| Family history of chronic arseniasis[c] | | | | |
| Blackfoot disease | | | | |
| No | 1021 | 60 | 5.88 | 1.00 |
| Yes | 56 | 6 | 10.71 | 1.54 (0.55–4.32) |
| Skin cancer | | | | |
| No | 1068 | 65 | 6.09 | 1.00 |
| Yes | 9 | 1 | 11.11 | 4.05 (0.44–37.27) |

[a]In 67 subjects there were no data on liver function test, or on hepatitis B surface antigen carrier status, and in four subjects there were no data on family history of chronic arseniasis. [b]Liver dysfunction was defined as an ALT ⩾ 50 U $l^{-1}$. [c]In four subjects there were no data on occupational exposure and lifestyle characteristics. *$P<0.05$.

**Table V** Multiple logistic regression analysis of risk factors associated with skin cancer among 1081 residents in blackfoot disease hyperendemic villages

| Variable | Group | Multivariate-adjusted odds ratio (95% confidence interval) |
|---|---|---|
| Age | Every one year increment | 1.12 (1.08–1.17)* |
| Sex | Women | 1.00 (referent) |
| | Men | 2.01 (1.08–3.75)* |
| Cumulative arsenic exposure (p.p.m.-years) | ⩽4 | 1.00 (referent)† |
| | 5–24 | 6.69 (0.76–59.17)[a] |
| | ⩾25 | 9.05 (1.06–77.27)* |
| Chronic hepatitis B carrier and liver function status | | |
| Non carrier with normal liver function | | 1.00 (referent) |
| HBsAg carrier with normal liver function | | 0.95 (0.52–2.16) |
| Non-carrier with liver dysfunction | | 2.73 (0.69–10.88) |
| HBsAg carrier with liver dysfunction | | 6.61 (1.75–25.03)* |
| History of working in a salt field | No | 1.00 (referent) |
| | Yes | 2.06 (1.01–4.18)* |
| Duration of consuming dried sweet potato (years) | ⩽9 | 1.00 (referent)† |
| | 10–19 | 5.46 (0.65–45.92)[a] |
| | ⩾20 | 8.54 (1.08–67.54)* |

[a]$0.05<P<0.1$. *$P<0.05$. †$P<0.05$ for trend test.

multivariate-adjusted odds ratio of developing skin cancer for each risk factor. Table V illustrates the results of the multiple logistic regression analysis. For every 1 year increment in age, there was a 1.12-fold increase in risk of skin cancer. Men had a prevalence of skin cancer approximately twice that for women. Cumulative arsenic exposure index was positively associated with the prevalence of skin cancer in a dose-response relation. Chronic HBsAg carriers with liver dysfunction had an increased risk of skin cancer, and a history of working in a salt field was also significantly associated with skin cancer. The duration of consuming dried sweet potato as a staple food was positively associated with the prevalence of skin cancer in a dose-response relation.

### Discussion

Since Hutchinson (1887) first reported the possibility that medication with inorganic arsenic is an aetiological factor for skin cancer, several types of neoplastic change of the skin, including Bowen's disease and basal and squamous cell carcinomas, have been associated with chronic arsenic exposure. According to previous ecological studies, the risk of skin cancer is significantly related to an increase in arsenic exposure among residents in BFD endemic areas (Tseng, 1977; Chen *et al.*, 1985 1988a, 1992; Wu *et al.*, 1989; Cheng and Wang, 1990). These studies may be subject to the ecological fallacy, and the association observed at the village level may not hold at the individual level. This study, performed with the specific aim of examining the association between arsenic and skin cancer at the individual level, found a dose-response relation beween chronic arsenic exposure and prevalence of skin cancer similar to that reported previously, before the tap water supply system was implemented (Tseng *et al.*, 1968).

However, there are some limitations in this study. Not all eligible study subjects participated in the health examination. As the frequency distribution of age, sex, educational level, occupation, lifestyle variables, personal and family history of disease and chronic arsenic exposure was similar between participants and non-participants, it seems unlikely that the non-response may confound the association observed between arsenic exposure and skin cancer. Because arsenic levels in drinking water in some areas other than the area endemic for BFD were not always available, the average and cumulative arsenic exposure levels were unknown for about one-quarter of study subjects. The duration of consuming artesian well water was similar for subjects with the information on arsenic exposure and those without it. Furthermore, the odds ratio for those without arsenic exposure levels was between the odds ratios for the lowest and highest exposure groups. The unavailability of arsenic exposure data for some study subjects seems not to affect the assessment of the association observed.

The prevalence rather than incidence of skin cancer was studied in this report. As skin cancer patients were documented to have an increased mortality from ischaemic heart disease and internal cancers than those who were not affected, the prevalence of skin cancer and the relative risk observed for each risk factor may be underestimated in this study. Most study subjects had been exposed to arsenic in drinking water since birth, and stopped drinking artesian well water in the early 1970s. It is hypothesized that the latent period from the exposure to ingested inorganic arsenic to the development of skin cancers may be as long as two to three decades; skin cancer may occur even years after cessation of the exposure. No skin cancer case was observed among men and women aged less than 45 years. They may have an arsenic exposure too low to induce the skin cancer. The prevalence of skin cancer was found to increase with age. Old age may reflect a high cumulative arsenic exposure, an inherent susceptibility to skin cancer resulting from ageing or both. After adjusting for the effect of cumulative arsenic exposure index, age remained a significant predictor, suggesting a possible role of ageing in the determination of skin cancer. The higher prevalence of skin cancer in men than women might be because men drink more than women or men may be more susceptible to skin cancer than women.

The consistent finding of a dose-response relation between arsenic exposure and skin cancer in this and previous studies makes it reasonable to conclude that arsenic is skin carcinogenic in humans despite inadequate evidence in animals (World Health Organization, 1980). Arsenic has been well documented to induce chromosomal aberrations and sister chromatid exchanges in human and rodent cells *in vitro* (Jacobson-Kram and Montalbano, 1985), to transform Syrian hamster embryo cells (Lee *et al.*, 1985) and to induce gene amplification (Lee *et al.*, 1988). However, arsenic does not induce mutation in prokaryotic and eukaryotic cells *in vitro* (Jacobson-Kram and Montalbano, 1985). Because arsenic has been reported to be neither an initiator nor a promoter in the two-stage model of animal carcinogenesis, it has been hypothesised to play a role in the progression phase of carcinogenicity (Lee *et al.*, 1988). Arsenic might induce human cancers by the induction of cell proliferation through its inhibition of thiol-dependent enzyme systems.

A positive association between sunlight exposure and skin cancer has been reported previously (Viyasa *et al.*, 1990; Green and Battistutta, 1990), but no association between sunlight exposure and arsenic-induced skin cancer was observed in this study. Further analysis on the combination of sunlight exposure and inorganic arsenic exposure did not shown any modifying effect on skin cancer for sunlight exposure. The lack of association with sunlight exposure may be due to the crude measurement of cumulative lifetime sunlight exposure. The misclassification of the exposure may result in the underestimation of odds ratios. Arsenic-induced skin cancer lesions have similar histological features to sunlight-related skin cancers (Deng and How, 1977), but their distributions of skin lesions are different. Arsenic cancers spread on every part of the body, while skin lesions induced by other carcinogens such as UV radiation and polyaromatic hydrocarbons are limited to the sites of exposure. In other words, arsenic-induced skin cancer lesions can be distinguished from those resulting from exposures to other skin carcinogens by their distribution on the body. The body site distribution of skin cancer was similar among men and women in this study. The wide spread of skin lesions induced by arsenic exposure might be due to a wide distribution of ingested arsenic in human tissues.

No association with skin cancer was observed for the habits of cigarette smoking and alcohol drinking in this study, but a history of working in salt fields was significantly correlated with an increased risk of skin cancer. Working under sunlight in a humid subtropical area, salt field workers tend to drink more water than those who are engaged in other occupations. This might explain the higher risk of arsenic-induced skin cancer among salt field workers.

Inorganic arsenic is biotransformed in the body to monomethylarsonic acid (MMA) and dimethylarsinic acid (DMA) (Tam *et al.*, 1979). Methylation of arsenic is considered to be a detoxication mechanism because DMA has a low acute toxicity (Vahter and Marafante, 1983) and is rapidly excreted in the urine (Vahter *et al.*, 1984). Although the complete mechanism of methylation of inorganic arsenic has not been elucidated, it seems clear that it takes place in the liver and is enzymatically mediated. Liver insufficiency (glutathione depletion) significantly modifies the ratio of the methylated metabolites excreted in urine (Buchet *et al.*, 1984), so that liver disease may have an affect on the methylation of arsenite in humans (Buchet *et al.*, 1984) or in rats (Buchet and Lauwerys, 1987). In the present study, we found a significantly increased prevalence of skin cancer among chronic HBsAg carriers with liver dysfunction. It is porposed that chronic HBsAg carriers with liver dysfunction would influence methylation and thereby detoxication of inorganic arsenic. Therefore, arsenic may be accumulated in the keratin-rich tissues such as skin, hair and epithelium of the upper gastrointestinal tract. Residents in BFD endemic areas were found to have a very poor nutritional status

(Blackwell, 1961). Dehydrated sweet potato and/or rice were the staple foods for people in Taiwan before 1960. Those who had a low socioeconomic status consumed much more dehydrated sweet potato than those with a high socioeconomic status. Our previous study has shown that the higher the consumption of dehydrated sweet potato, the lower the intake of other nutrients and the poorer the nutritional status (Chen *et al.*, 1988b). In this study, we found a dose-response relation between the prevalence of skin cancer and the duration of consumption of dried sweet potato as a staple food, which was used as an indicator of undernourishment. It has been reported that subjects with a poor nutritional status have a lower capacity for methylation and arsenic detoxication (Vahter and Marafante, 1987). An intervention study on arsenic methylation among people who are using high-arsenic drinking water is recommended for the further validation of these findings.

**Acknowledgements**
This work was supported by the National Science Council, Executive Yuan, Republic of China (NSC-80-0412-B-002-17, NSC-81-0412-B-002-122, NSC-82-0412-B-002-262).

**References**

BLACKWELL RQ. (1961). Estimated total arsenic ingested by residents in the endemic blackfoot area. *J. Formosan Med. Assoc.*, **60**, 1143.

BROWN SM AND RABINOWITZ AD. (1979). The precursors of cutaneous squamous cell carcinoma. *Int. J. Dematol.*, **18**, 1-16.

BUCHET JP AND LAUWERYS R. (1987). Study of factors influencing the in vivo methylation of inorganic arsenic in rats. *Toxicol. Appl. Pharmacol.*, **91**, 65-74.

BUCHET JP, GEUBEL A, PAUWELS S, MAHIEU P AND LAUWERYS R. (1984). The influence of liver disease on the methylation of arsenite in human. *Arch. Toxicol.*, **55**, 151-154.

CEBRIAN ME, ALBORES A, AGUILAR M AND BLAKELY E. (1983). Chronic arsenic poisoning in the north of Mexico. *Hum. Toxicol.*, **2**, 121-33.

CHEN KP AND WU HY. (1962). Epidemiologic studies on blackfoot disease: a study of source of drinking water in relation to disease. *J. Formosan Med. Assoc.*, **61**, 611-617.

CHEN CJ AND WANG CJ. (1990). Ecological correlation between arsenic level in well water and age-adjusted mortality from malignant neoplasms. *Cancer Res.*, **50**, 5470-5474.

CHEN CJ, CHAUNG YC, LIN TM AND WU HY. (1985). Malignant neoplasms among residents of a blackfoot disease endemic area in Taiwan: high-arsenic artesian well water and cancers. *Cancer Res.*, **45**, 5895-5899.

CHEN CJ, KUO TL AND WU MM. (1988*a*). Arsenic and cancers. *Lancet*, **ii**, 414-415.

CHEN CJ, WU MM, LEE SS, WANG JD, CHENG SH AND WU HY. (1988*b*). Atherogenicity and carcinogenicity of high-arsenic artesian well water: multiple risk factors and related malignant neoplasms of blackfoot disease. *Arteriosclerosis*, **8**, 452-460.

CHEN CJ, CHEN CW, WU MM AND KUO TL. (1992). Cancer potential in liver, lung, bladder and kidney due to ingested inorganic arsenic in drinking water. *Br. J. Cancer*, **66**, 888-892.

DENG JS AND HOW SW. (1977). Comparison of the fine structure of basal cell carcinoma in ordinary patients and patients with chronic arsenicalism. *J. Formosan Med. Assoc.*, **76**, 685-692.

FROST DV. (1967). Arsenicals in biology: retrospect and propect. *Fed. Proc.*, **26**, 194-208.

GREEN A AND BATTISTUTTA D. (1990). Incidence and determinants of skin cancer in a high risk Australian population. *Int. J. Cancer*, **46**, 356-361.

HUTCHINSON J. (1987). Arsenic cancer. *Br. Med. J.*, **2**, 1280-1281.

INTERNATIONAL AGENCY FOR RESEARCH ON CANCER (1987). Monographs on the evaluation of carcinogenic risks to humans overall evaluations of carcinogenicity: an updating of IARC Monographs, Vol 1-42, Suppl. 7. IARC: Lyon.

JACOBSON-KRAM D AND MONTALBANO D. (1985). The reproductive effects assessment group's report on mutagenicity of in organic arsenic. *Environ. Mutagenesis*, **7**, 787-804.

KUO TL. (1964). Arsenic content of artesian well water in endemic area of chronic arsenic poisoning. *Reports, Institute of Pathology, National Taiwan University*, **20**, 7-13.

LEE TC, OSHIMURA M AND BARRETT JC. (1985). Comparison of arsenic-induced cell transformation, cytotoxicity mutation and cytogenetic effects in syrian hamster embryo cells in culture. *Carcinogenesis*, **6** (9-12), 1421-1426.

LEE TC, TANAKA N, LAMB PW, GILMER TM AND BARRETT JC. (1988). Induction of gene amplification by arsenic. *Science*, **241**, 79-81.

LO MC, HSEN YC AND LIN BK. (1977). Arsenic content of underground water in Taiwan: second report. Taiwan Provincial Institute of Environment Sanitation: Taichung, Taiwan.

NEUBAUER O. (1947). Arsenic cancer: a review. *Br. J. Cancer*, **1**, 192-251.

NELSON WC, LYKINS MH, MACKEY J, NEVILL VA, FINKLEA JF AND HAMMER DI. (1973). Mortality among Orchard workers exposed to lead arsenate spray: a cohort study. *J. Chronic Dis.*, **26**, 105-118.

ROTH F. (1957). Concerning the delayed effects of the chronic arsenicism of the Moselle wine growers. *Deutsche Medizinische Wochenschrift*, **82**, 211-217.

SOMMERS SC AND McMANUS RG. (1953). Multiple aresenic cancers of skin and internal organs. *Cancer*, **6**, 347-359.

TAM GKH, CHARBONNEAW SM, BRYCE F, POMROY C AND SANDI E. (1979). Metabolism of inorganic arsenic (74As) in humans following oral ingestion. *Toxicol. Appl. Pharmacol.*, **50**, 319-322.

TSENG WP. (1977). Effects and dose-response relationships of skin cancer and blackfoot disease with arsenic. *Environ. Health Perspect*, **19**, 9-19.

TSENG WP, CHU HM, HOW SW, FONG JM, LIN CS AND YEH S. (1968). Prevalence of skin cancer in an endemic area of chronic arsenicism in Taiwan. *Journal of the National Cancer Institute*, **40**, 453-463.

VAHTER M AND MARAFANTE E. (1983). Intracellular interaction and metabolic fate of arsenite and arsenate in mice and rabbits. *Chem. Biol. Interact.*, **47**, 29-44.

VAHTER M AND MARAFANTE E. (1987). Effect of low dietary intake of methionine, choline or proteins on the biotransformation of arsenite in the rabbit. *Toxicol. Lett.*, **37**, 41-46.

VAHTER M, MARAFANTE E AND DENCKER, L. (1984). Tissue distribution and retention of 74As-dimethyl-arsenic acid in mice and rats. *Environ. Contam. Toxicol.*, **13**, 259-264.

VIYASA BC, TAYLOR HR, STRICKLAND PT, ROSENTHAL FS, WEST S, ABBEY H, NG SK, MUNOZ B AND EMMETT EA. (1990). Association of non-melanoma skin cancer and actinic keratosis with cumulative solar ultraviolet exposure in Maryland watermen. *Cancer*, **65**, 2811-2817.

WORLD HEALTH ORGANIZATION (1980). IARC monographs on the evaluation of the carcinogenic risk of chemical to humans: some metal and metallic compounds. International Agency for Research on Cancer: Lyon.

WU HY, CHEN KP, TSENG WP AND HSU CL. (1961). Epidemiologic studies on blackfoot disease. I. Prevalence and incidence of the disease by age, sex, year occupation and geographical distribution. *Mem. College Med. Natl: Taiwan Univ*, **7**, 33-50.

WU MM, KUO TL, HWANG YH AND CHEN CJ. (1989). Dose-response relation between arsenic concentration in well water and mortality from cancer and vascular disease. *Am. J. Epidemiol.*, **130**, 1123-1132.

YEH S. (1973). Skin cancer in chronic arsenicism. *Hum. Pathol.*, **4**, 469-485.

YEH S, HOW SW AND LIN CS. (1968). Arsenical cancer of skin. *Cancer*, **21**, 312-329.